# Supplementary material for: The interactome of intact mitochondria by cross-linking mass spectrometry provides evidence for coexisting respiratory supercomplexes
Source: Mol Cell Proteomics. 2017 Dec 8;17(2):216–32. doi: 10.1074/mcp.RA117.000470 (PMC5795388; doi:10.1074/mcp.RA117.000470)
Supplement: Supplemental Data [file supp_RA117.000470_133922_0_supp_23978_fzffwf.zip › spectra_annotation/mito_DR_spectra_annotation/303-1-3-1-4-1.pdf]

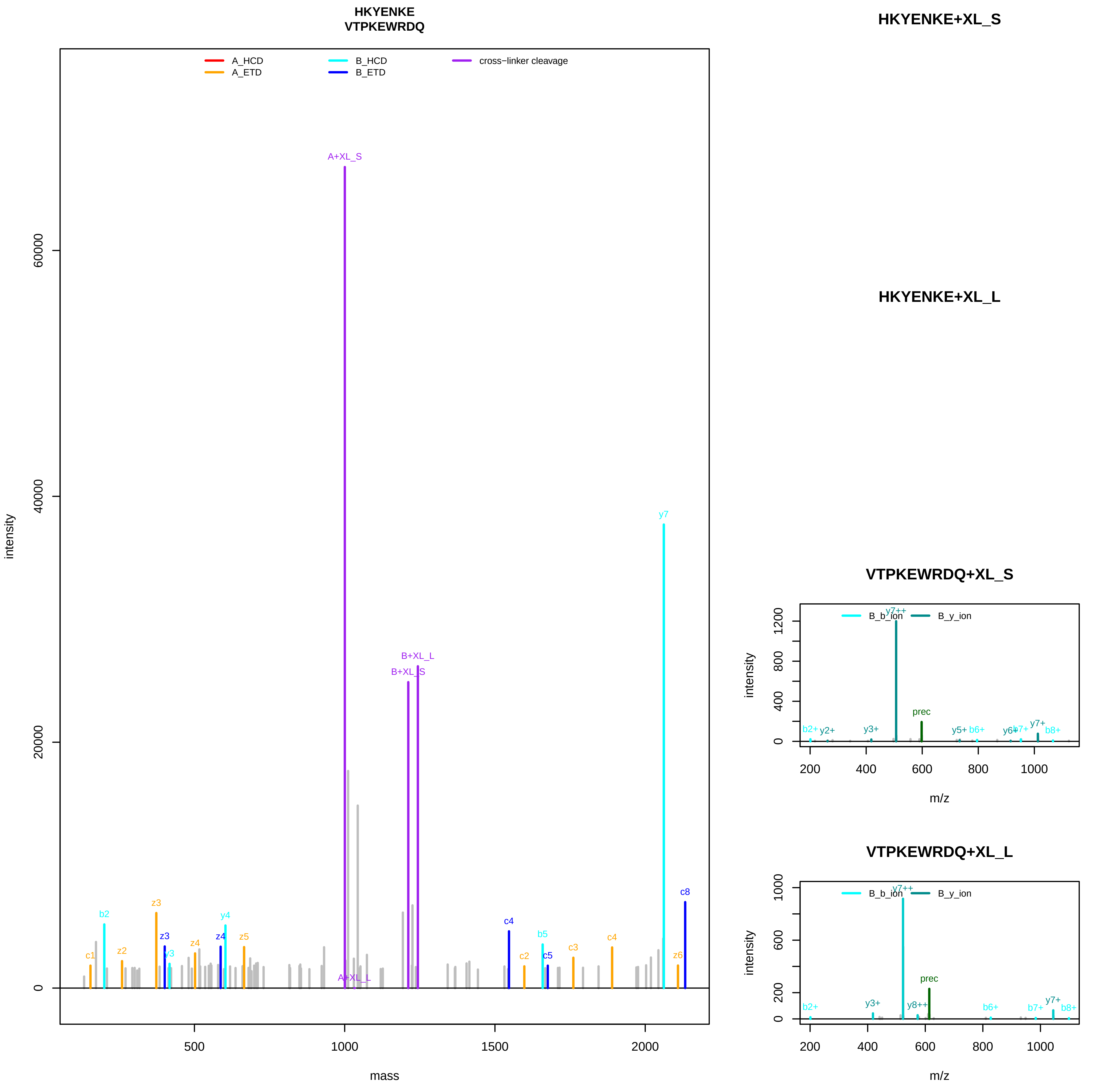

HKYENKE
VTPKEWRDQ
A_HCD
A_ETD
B_HCD
B_ETD
cross−linker cleavage
intensity
mass
0
20000
40000
60000
500
1000
1500
2000
A+XL_S
A+XL_L
B+XL_S
B+XL_L
y7
c8
c1
b2
z2
z3
z3
y3
z4
z4
y4
z5
c4
c2
b5
c5
c3
c4
z6
HKYENKE+XL_S
HKYENKE+XL_L
VTPKEWRDQ+XL_S
B_b_ion
B_y_ion
y7++
prec
b2+
y2+
y3+
y5+
b6+
y6+
b7+
y7+
b8+
intensity
m/z
0
400
800
1200
200
400
600
800
1000
VTPKEWRDQ+XL_L
B_b_ion
B_y_ion
y7++
prec
b2+
y3+
y8++
b6+
b7+
y7+
b8+
intensity
m/z
0
200
600
1000
200
400
600
800
1000
